# University of Michigan and the Peninsular Journal

**Published:** 1855-11

**Authors:** 


*The Medical Department of the University of Michigan and the Peninsular Journal, again.*—Naturalists tell us that there is a species of fish, which possesses the faculty, when pursued by an enemy, of ejecting a considerable quantity of a black fluid for the purpose of coloring the water and thereby hiding itself from its pursuer.

There is a class of writers who always remind us of this fish. If they engage in controversy, instead of proceeding directly to a discussion of the question involved, they are continually endeavoring to render the *waters* turbid; in other words, constantly hideing themselves or their real positions behind a mass of mere verbiage about side-issues and personal matters. One of the most common methods of doing this, is to make frequent professions of courtesy, of great forbearance, of being *compelled* to do thus and so; while their opponents are studiously represented as not only destitute of all these qualities, but wantonly unfair and ferocious in their assaults. By pursuing such a course the controversialist not merely aims to obscure his own positions but also to win for himself, as an *accused* party, the *sympathy* of his readers. We have been forcibly reminded of these thoughts by an editorial in the October number of the *Peninsular Journal of Medicine.* Our readers will remember that a paragraph in the May number of this Journal, called out an editorial in the July number of the *Peninsular Journal*, to which we replied in our September number. The simple question at issue was, whether the Medical Department of the University of Michigan was actually "*so far in advance of almost every other school in the country, in the cause of reform,*" *pointed out* in Dr. Cabell's report on medical education, published in the *Transactions* of the American Medical Association? To enable our readers to determine that question for themselves, in our article in the September number, we expressly refused to reply to any personal allusions or side issues, and proceeded directly to quote Dr. Cabell's views in his own words, together with the previous recommendations of the Association to which he referred. We also quoted *verbatim*, the requirements of the Michigan School, as published in the last annual announcement, thereby enabling each reader to judge for himself concerning its *advances* and delinquencies.

In closing our article we alluded to a comparison, instituted between the Michigan School and the Rush Med. College; characterizing it as "unfair and uncalled for," at the same time quoting the part complained of, and adding the assertion, that "in the *position in which it was placed* by its author, it conveys the most glaring falsehoods." In reply to this, we have in the October number of the *Peninsular Journal*, *twelve* pages of what the editor, Dr. A. B. Palmer, calls a "defense." It commences with the usual professions of "editorial courtesy," of "fairness," of "forbearance even," and of the "greatest reluctance" on his own part; while he attributes to our humble self "such an array of unmanly evasions, untruthful statements, false accusations, and bitter bursts of feeling;" of having used "false and abusive language;" of being "under some strange infatuation—some *demon* of *passion*," of being his "unjust accuser—nay *his* bitter traducer;" and such a host of other similar expressions, that those of his own subscribers who read only his articles on the subject, would almost conclude that our editorial in the September number, had emanated from the very fountain of overflowing passion, and been written with a pen literally dipped in *gall* and *wormwood*.

If this style of writing is in accordance with the *taste*—the sense of propriety, of our amiable confrére of the *Peninsular Journal*, we will not dispute with him about it; but he shall continue to have the whole field, in that department, to himself.

And as he has found no "space," in the whole of his twelve pages, to make even the pretense of a reply to the facts we adduced in our former article, we will notice only a few additional items at this time. First, in regard to the main question at issue, the editor of the *Peninsular Journal* emphatically denies ever having claimed that the Michigan School had *fully* complied with all the requirements of the National Association.

He says, "now we repeat, as it is essential to be understood that the only issue we made was in relation to the specific measures in Dr. Cabeli's report. We have never said that in *each minute particular* the University of Michigan had complied with the recommendations of the National Association, though in the greater matters it has." We hope he will remember this admission, and when he publishes in the next Annual Announcement that "the *peculiar* position" of the Michigan University "is such that it has been enabled *fully to comply* with the demands of the profession;" or in his next speech before the National Association, he speaks of it as "an institution which had sought to *comply* with the *recommendations* of that body;" and the founders of which, "sought in its organization to follow your (the Association's) directions," he will be careful to specify those "minute particulars" to which he alluded. Because there might be much difference of opinion about the relative *minuteness* of some of the items, such as the omission of clinical instruction, the failure to require personal attention to dissections for a reasonable time, the deduction of *one year* from the ordinary period of study, &c. At present, however, we will so far gratify the editor of the *Peninsular Journal*, as to limit our further comments to those "*greater matters*," concerning which he *now* claims that the University is "so far in advance of almost every other school in the country."

We will first quote them in his own words as follows:

"The great principles of a proper standard of preliminary education—a long term of lectures, with thorough daily examinations in connection; and a high standard of professional attain ments requisite to graduation. The University of Michigan *does profess* in these *great matters*, to have taken a step *in advance* of the other schools." Here we certainly have a distinct claim to superiority set up in behalf of that school, in regard to four well defined items, viz.; a high standard of professional attainments; thorough daily examinations; a long term of lectures; and a proper standard of preliminary education. Is this claim well founded? Is there no "*baseless pretention*," lurking in it? We would like to have the editor of the *Peninsular Journal*, specify in what respect the University of Michigan requires of its candidates for graduation, a *higher* standard of *professional* attainments than the other medical schools. Does the Faculty of that school require of its candidates a knowledge of any branch of

Medical Science not required by other medical schools? Certainly not, but on the contrary it wholly omits the important departments of clinical medicine and surgery, which very many of the other schools require Does the Faculty of that school require of its candidates a longer period of medical study than other schools? So far from this, it allows a part of them to deduct *one third* from the usual period of study required by every other institution with which we are acquainted. Is it not plain then, that the University of Michigan, instead of having "taken a *step in advance* of the other schools" in regard to a higher standard of professional attainments, has actually retrograded both by narrowing the *field* of attainments and shortening the *time* required for its cultivation?

The claim set up to superiority in the matter of "daily examinations," rests on an equally slender foundation. The practice of making such examinations, and making them thoroughly, too, is neither new to the medical colleges of our country, nor by any means peculiar to the University of Michigan. Full *twenty* years ago, when Dr. Palmer and myself were both attending the same Medical College, the different members of the Faculty practiced as thorough daily examinations, as have ever been practiced by our neighbors in Michigan. And the same has been done in a large portion of the medical colleges in this country from that time to the present. When in our former article it was stated that all the members of the Faculty of Rush Medical College made it a rule to practice daily examinations of their classes, the word *rule* was used, not, as represented by Dr. Palmer, for purposes of *evasion*, but simply to express a fact. The rule is to examine daily; but sometimes when one subject requires several lectures for its full consideration, the examinations are deferred until the subject is completed and then time enough is devoted to review the whole by one examination.

In some of the best colleges in the country, the different members of the Faculty, instead of questioning the class daily on the subject of the lecture which has preceded, set apart one or two hours each week which they devote exclusively to thorough and systematic examinations.

Such has been the case in the college of physicians and surgeons of New York. For the editor of the *Peninsular Journal* to dignify these daily examinations with a place among the "*greater matters*" pertaining to " medical reform," and then to assume that himself and colleagues in the University of Michigan attend to them so much more thoroughly than all other medical teachers as to constitute a " step in advance," is not what we will call *arrogance*; for that would probably "*astonish*" him again beyond measure. But we will leave each reader to choose for it a term to suit himself. The next *great* matter concerning which the University of Michigan claims to have taken " a step in advance of the other schools," is the adoption of a *long* lecture term; extending to a little more than six months of the year. But, as we stated in our former number, even this is not really a "*step in advance*" of all the other schools in the country, either in regard to the time of its adoption, or its absolute length. Several years before the medical department of the University of Michigan had an existence, the medical department of the University of Virginia adopted a lecture *term* embracing *nine* months of the year, and has continued it up to the present time. So did the University of Pensylvannia and the College of Physicians and Surgeons of New York, adopt a lengthened lecture term [the one six, and the other five months], before the Michigan School possessed an active existence. So far then from having taken a step in advance, the latter school by adopting a long lecture term, has only *followed* in the footsteps of some of the oldest medical colleges on this side of the Atlantic. We would not detract in the least from any credit which is justly due our Michigan friends, for having adopted a long lecture term. It was only their boast of being " so far in advance of almost every other in the country," that we objected to as groundless.

Since the claim to being a step in advance of the other schools in this respect has been repeated with so much emphasis by the editor of the *Peninsular Journal*, it may be well to enquire briefly, whether the lengthened term itself has been used in such a manner as to accomplish the important purposes for which a long term was demanded by the profession. The great leading purpose for which a longer lecture term was required, was, that the Faculties connected with the several medical schools, might so enlarge the curriculum of medical studies as to embrace more fully the important departments of General, Microscopic and Practical Anatomy, together with Clinical Medicine and Surgery. Instead, however, of devoting their lengthened term to the accomplishment of these important objects, our Michigan cotemporaries, have simply used the additional time to drill their candidates for graduation, once in from two to four weeks, in the ordinary grammar school exercise of writing and reading compositions. How far the profession generally will regard this as a step in advance, we will not pretend to decide.

The *fourth* and last of the "great matters," concerning which the Michigan University claims to have taken a step in advance of the other schools, consists in the requirement of "a proper standard of preliminary attainments." This indeed, may be regarded as the chief theme of their boasting and pride. It was *the* topic on which Dr. Cabell, in his report made allusion to the University. And certainly so far as they actually require any just standard of *preliminary education*, we will most cheerfully give them all due credit and honor. For when Dr. Palmer says in his last article that we have "*now ignored*," i. e., repudiated the importance, of this subject he makes an assertion without the shadow of a foundation. So far from this, in our article in the September number, we quoted fully all the requirements they have on the subject, and also enumerated it as almost the only item they had left on which to found a claim to advancement beyond other schools. But what is the exact position of the University of Michigan on this subject; and to how much credit are they entitled therefor? Their last Annual Announcement says: "each candidate for admission must be provided with satisfactory evidence of good moral character; and if a candidate for graduation, 'also of such literary attainments as have been recommended by the American Medical Association.' This, though rather indefinite is good as far as it goes; but in the same Announcement and almost in the same paragraph we have the following, viz.: "To encourage a higher grade of *preliminary acquirement*, an allowance of *one year* from the term of study is made in favor of graduates of the Colleges of Science and Arts, and of other respectable literary colleges." The inconsistency of these provisions is very obvious. If the first is in good faith designed to secure on the part of all, an adequate amount of preliminary attainments, such as has been recommended by the National Organization, then the offer made in the second to deduct *one third* from the already restricted period of medical study "*to encourage*" the same thing, is entirely uncalled for, and highly mischievous in its tendency. How much credit should be awarded to them for taking "a step in advance" on the subject of preliminary education, while they allow so large a deduction from the regular course of study, we leave for the profession generally to decide. That part of our former article which seems to have given our friend, the editor of the *Peninsular Journal*, the greatest offense was in relation to his comparison between the requirements of the Michigan school and Rush Medical College. We alledged that his comparison was *unfair*, and that a portion of the language used, "in the position in which it was placed by its author, conveyed the most glaring falsehoods;" at the same time quoting in full the paragraph to which we alluded. This Dr. Palmer, styles an "outrageous accusation," and on the strength of it, tells his readers that we have charged him in terms, of uttering the most glaring falsehoods." In making this allegation, the Doctor has done himself, as well as us, decided injustice. He would make his readers believe we had charged him with *wilful lying*; whereas, our distinct accusation was that of *unfairness*, which consisted in his having used language that "in the connection in which it was placed" would convey to the mind of the reader the most erroneous, or false impressions, or "*falsehoods*;" the latter word being used as strictly synonymous with *errors*. But Dr Palmer, goes further and says: "now our neighbor, in his eagerness to convict us of untruth, and give excuse for his original unfounded accusation, has most dishonorably, it seems to us, concealed the fact that we were speaking *exclusively* on the subject of preliminary education—has garbled our paragraph, leaving out the sentences which so clearly show it, quoting only a part, which, taken out of connection seems to mean a different thing." Here is a distinct claim that in the comparison, of which the paragraph we quoted was a part, he was speaking exclusively on the subject of preliminary education; and in another place he adds: "all that bears upon that subject we did mention—beyond that we did not pretend to go."

That our readers may judge concerning the *truth* of this last assertion, together with the extent of our previous "concealment" and "garbling," we will quote the whole of the original comparison alluded to. And here it is:

"The first reform urged in Dr. C.'s report, is the exacting of a higher standard of preliminary education of medical students.

"*On this point*, Rush Medical College exacts nothing of one who joins their class, but the registration of his or her name, and the payment of the ticket fees, if he or she attends the lectures. *That institution admits to the graduating class all who have studied the usual time, attended two courses of lectures of sixteen weeks, and who write and hand in a single thesis, which is seldom, perhaps, read—never by the student before the faculty or class.*"

"The University of Michigan feels at present under obligation to admit to the *lectures* all who possess a good moral character, and pay the matriculation fee of ten dollars. Those however who are admitted as candidates for *graduation*, must present themselves at the *commencement* of their second course of lectures, or after four years of reputable practice—show that they have studied medicine the proper length of time—must either present clear evidence, by certificates from competent sources, that they have 'a good English education, a knowledge of natural philosophy, and the elementary mathematical sciences, including Geometry and Algebra, and such an acquaintance, at least, with ancient languages as will enable the student to appreciate the technical languages of medicine and read and write prescriptions;' or in case such evidence is not furnished, the candidate must submit to an examination on preliminary education by a committee of the faculty. The candidates for graduation must also at the beginning of the term pass a satisfactory examination in Anatomy, Chemistry, Physiology, and Materia Medica, and once in from two to four weeks during the entire term, must present to the Faculty, and read and defend before them and such of the class as choose to attend, a thesis on some medical subject, as well as present a final thesis, upon which they must pass a public and rigid examination, usually from half an hour to an hour or more in length. In all these written exercises, originality of composition, clearness of language and doctrine, and precision of thought are requisite, and imperfections are pointed out and commented upon. This ordeal of writing and public reading is a more thorough test of 'preliminary education' than can well otherwise be afforded."

The lines in italics, constitute the part which we quoted in our former article. Every reader will see that they constitute a complete independent sentence; the meaning of which is in no degree modified by any thing that precedes or follows it. Hence in quoting it, we neither "concealed" nor "garbled" anything whatever. It is true we omitted the heading of "preliminary education," and thereby, Dr. Palmer says, "we concealed the fact that the comparison related 'exclusively' to that subject; as though the *heading* or title must necessarily fix and limit the character of all that followed." To set up the claim that in the comparison above quoted, h e mentined "all that bears on" the subject of preliminary education, and nothing "beyond that," is surely the weakest of all pretexts. Will he tell us what *bearing* the payment of the "ticket fees," the "usual time" of medical study, the number and length of the courses of lectures, the time of previous practice, and the time of making the examinations in Anatomy, Chemistry, &c., have on the subject of preliminary education?

They are all topics occupying a prominent position in his comparison; and when he so blusteringly attempts to convict us of garbling and dishonest concealment, by claiming that he wrote *nothing beyond* what related to preliminary education, we think the "utter astonishment" which he speaks of in the commencement of his article must have seriously confused his ideas. But we are bestowing more time and ink on the article of our cotemporary than its merits deserve; and hence will not stop to notice several unfair and erroneous statements. Being entirely willing to let the candid reader judge whether an institution, "placed by the munificence of the State," entirely above want, can justly proclaim itself, "in all the *greater matters*," a step in *advance* of the other Colleges, while it omits entirely from its requirements all attention to Clinical Medicine and Surgery, and in some cases permits the deduction of one whole year from the usual period of medical study.

